# A Review on Electroactive Polymers for Waste Heat Recovery

**DOI:** 10.3390/ma9060485

**Published:** 2016-06-17

**Authors:** Ewa Kolasińska, Piotr Kolasiński

**Affiliations:** 1 Electrotechnical Institute, Division of Electrotechnology and Materials Science, Marii Skłodowskiej-Curie 55/61, Wrocław 50-369, Poland; 2 Department of Thermodynamics, Theory of Machines and Thermal Systems, Faculty of Mechanical and Power Engineering, Wrocław University of Science and Technology, Wybrzeże Wyspiańskiego 27, Wrocław 50-370, Poland; piotr.kolasinski@pwr.edu.pl

**Keywords:** thermoelectric materials, electroactive polymers, waste heat recovery, energy efficiency, innovative energy conversion systems

## Abstract

This paper reviews materials for thermoelectric waste heat recovery, and discusses selected industrial and distributed waste heat sources as well as recovery methods that are currently applied. Thermoelectric properties, especially electrical conductivity, thermopower, thermal conductivity and the thermoelectric figures of merit, are considered when evaluating thermoelectric materials for waste heat recovery. Alloys and oxides are briefly discussed as materials suitable for medium- and high-grade sources. Electroactive polymers are presented as a new group of materials for low-grade sources. Polyaniline is a particularly fitting polymer for these purposes. We also discuss types of modifiers and modification methods, and their influence on the thermoelectric performance of this class of polymers.

## 1. Introduction

Since environmental and energy efficiency regulations are becoming increasingly strict (see e.g., [1,2]) there is a need for research on renewable energy conversion systems that will reduce the greenhouse gases emission and improve energy efficiency. Effective and rational waste heat recovery from industrial and distributed sources can increase the market share of renewable energy systems and, in turn, improve energy efficiency and reduce fossil fuel consumption. However, implementing effective waste heat recovery requires the relevant energy conversion technologies. Thus, research on methods of waste heat conversion into electricity and other useful forms of energy is important. 

This paper reviews the thermoelectric materials suitable for waste heat recovery. Section 2 discusses the waste heat sources and presents recovery technologies. Section 3 provides information about thermoelectric effects and thermoelectric parameters. The following sections of this paper discuss the literature on the thermoelectric properties of various thermoelectric materials, *i.e.*, electrical conductivity, thermopower, thermal conductivity and thermoelectric figures of merit. Section 4 briefly reviews research on inorganic alloy- and oxide-based thermoelectric materials suitable for heat recovery from medium- and high-grade waste heat sources. The literature shows that electroactive polymers, in particular polyaniline-based materials, have the potential for direct waste heat recovery from low-grade sources, and are comprehensively reviewed in Section 5. This summary provides insight into the directions of future research on this topic.

## 2. Waste Heat Sources and Current Recovery Technologies

Waste heat sources may differ in quality, thermal power and temperature range [3,4,5]. They can be classified by thermal power into the following categories [3]: large-power (more than 500 kW_t_), medium-power (10–500 kW_t_), small- and micro-power (0.5–100 kW_t_). By temperature range, waste heat sources can be classified into the following categories: high-grade (500–1500 °C), medium-grade (250–500 °C) and low-grade (40–250 °C). Table 1 provides information about the temperature range of the selected waste heat sources and most common waste heat carriers. The comparison is based on information reported in references [3] and [5].

The comparison in Table 1 shows gases (exhaust gases, waste steam, cooling air, *etc.*) or liquids (cooling mediums, hot liquids, *etc.*) are waste heat carriers. Waste heat is also dissipated from hot solid surfaces (boilers, heat exchangers, machines, pipelines, *etc.*). A major part of high- and medium-grade waste heat is generated in heavy industry (power and cement plants, paper-mills, steel-mills, glass-works, petroleum refineries, chemical plants, food processing plants). These are mainly large- and medium-power sources [4]. Low-grade waste heat is generated both in industry and in distributed energy generation systems and domestic appliances. Thus, low-grade waste heat sources are more common [4]. Regardless the temperature range, the major part of waste heat is emitted from the solid elements of installations and machinery, e.g., pipelines, machines casings.

Nowadays, numerous recovery technologies are available, and choosing among them is dependent mostly on temperature and the thermal power of the waste heat source. PCM-based (Phase Change Materials), heat storage systems have been proposed for high- and medium-grade waste heat storage. The overall efficiency of these devices is good and reaches 95% [3]. However, the use of stored heat may be difficult because most of the industrial plants deal with an excess, rather than a deficiency, of heat [4]. PCM-based heat storage systems are comprehensively described in references [6,7,8,9,10,11,12]. Various thermodynamic cycles such as the Striling, organic Rankine and Kalina cycles are currently applied for converting high- and medium-grade waste heat into electricity on an industrial scale. These technologies are suitable only to indirect waste heat recovery from liquids or gases. They are mechanically complicated and expensive, since heat exchangers, machines, and automatic control units must be employed. Moreover, they use hazardous low-boiling fluids as the heat-carrying agents. A large amount of power is needed for powering the feed-pumps and other devices. The overall efficiency of ORC (organic Rankine cycle) systems powered by high- and medium-grade waste heat varies in the range of 15%–20% [3]. The Kalina cycle is about 3% more efficient under the same operating conditions [3]. The overall efficiency of Stirling engines powered by high-grade waste heat reaches 30% [3]. The working principles of these systems and their applications are comprehensively treated in references [12,13,14,15,16,17,18,19,20,21,22,23,24,25,26,27,28,29,30,31,32,33,34,35,36,37,38,39,40]. Small- and micro-power Stirling engines and ORC systems have been considered for heat recovery from low-grade waste heat sources [16,25,26]. However, due to construction and design problems, most of these systems are still lab-prototypes or are being researched. The overall efficiency of ORC systems powered by low-grade waste heat varies in the range of 6%–9% [3], while the overall efficiency of Striling engine under the same operating conditions reaches 5%–6% [3]. These technologies require regular servicing, increasing their maintenance costs. Additionally, when the system fails, the value in lost production outweighs energy savings.

Technologies described above are not suitable for direct waste heat recovery from most common waste heat sources, *i.e.*, the solid elements of installations and machinery, and no relevant technologies are available. Thus, waste heat recovery from the solid elements is challenging. These elements can have various shapes and surfaces, such as flat, curved, cylindrical, spherical or finned. Thermoelectric materials may be used for harvesting waste heat from such components. They provide an opportunity for direct waste heat conversion into electricity without employing thermodynamic cycles, additional devices, moving parts, working fluids, and power supply. There is much research on alloy- and oxide-based thermoelectric materials suitable for medium- and high-grade heat sources. However, suitable materials for low-grade heat sources are still lacking. Electroactive polymers are promising for such applications.

## 3. Thermoelectric Effects and Thermoelectric Parameters

The thermoelectric materials, utilizing the Seebeck and Peltier effects, enable a direct conversion between heat and electricity. Thermoelectric energy conversion can be used to capture electric power from waste heat in a variety of applications [41]. A number of scientific studies and industrial research is focused on practical application of these materials for cooling and power generation [42,43,44]. The Seebeck effect occurs when the applied temperature gradient generates the current flow through the conducting material [45]. The Peltier effect occurs when a current flow through a conducting material results in heat generation or cooling [45,46,47]. These thermoelectric effects occur as a result of the interdependent processes of electric charge and heat transport [46,47,48]. The theoretical efficiency of the thermoelectric materials and devices is given by Equation (1):

(1) $$\eta =(\frac{T_{H}-T_{C}}{T_{H}})\frac{(\sqrt{1+ZT}-1}{\left(\sqrt{1+ZT}-(T_{C}/T_{H})\right)}$$

where *T_H_* (K) is the hot side temperature, *T_C_* (K) is the cold side temperature, and *ZT* is the thermoelectric figure of merit.

The dimensionless thermoelectric figure of merit *ZT* is given by Equation (2):

(2) $$ZT=\frac{\alpha^{2}\sigma T}{k}=\frac{\alpha^{2}T}{\rho k}$$

where *α* (V/K) is the Seebeck coefficient (also known as the thermopower), *σ* (S/m) is the electrical conductivity, *ρ* (Ω∙m) is the electric resistivity, *k* [W/m∙K] is the thermal conductivity, and *T* (K) is the absolute temperature [48,49]. The figure of merit in the form of *Z* (K^−1^) can be also found in the literature [50,51].

In reference [52], the development of the thermoelectric materials was divided according to *ZT* values into three following generations:

- The first generation materials with *ZT* about 1.0 and conversion efficiency of 4%–5%;
- The second generation materials (developed in 1990s) with *ZT* up to 1.7 and conversion efficiency of 11%–15%;
- The third generation materials (under development) with *ZT* up to 1.8 and predicted conversion efficiency of 15%–20%.

In reference [50], it is pointed out that the material with *ZT* ≈ 3 would achieve efficiency comparable to mechanical cooling technologies (e.g., mechanically driven fans or refrigeration cycles). Such thermoelectric material could be applied as a cooling device with the simultaneous function of being a power generator.

According to Equation (2), to obtain a high *ZT*, both Seebeck coefficient (*α*) and electrical conductivity (*σ*) should be large, but the thermal conductivity (*k*) should be low. Synthesis of such a material is still a challenge, due to an inconvenient coupling of the Seebeck coefficient with thermal and electrical conductivity limiting the value of *ZT* and thermoelectric efficiency [48,50].

## 4. Review of Inorganic Thermoelectric Materials for Waste Heat Recovery

Thermoelectric effects were first observed for metals [50,51,52]. However, their thermoelectric efficiency was too low for practical application. The efficiency of alloy-based materials is sufficient for thermoelectric application. The most important thermoelectric parameters of selected alloys and metals are reported in Table 2. The comparison, based on information reported in references [50] and [51], shows that metals are characterized by the highest values of electrical conductivity; however, their high thermal conductivity lowers the value of the Seebeck coefficient and the thermoelectric efficiency. Semiconducting alloy materials show lower thermal conductivity and higher Seebeck coefficient, which results in *ZT* ≈ 1. Their thermoelectric efficiency is thus higher than metals.

Bismuth telluride, bismuth antimonide as well as alloys of antimony, selenium, tellurium and bismuth are the commonly used thermoelectric materials, as the examples of Bi_2_Te_3_/Bi_2_Se_3_ (*ZT* = 0.6) and Bi_2_Te_3_/Sb_2_Te_3_ (*ZT* = 0.9) are mentioned [50]. Most of these materials exhibit the best thermoelectric efficiency at medium- and high-temperatures [42]. The working temperature of these alloy materials varies in ranges of <400 K for Bi_2_Te_3_, 600–900 K for PbTe and >900 K for SiGe [52].

The need of development materials and devices with improved thermoelectric efficiency leads to investigations on modification of the chemical composition as well as the design of thermoelectric generators. It is found that the conversion efficiency of the thermoelectric generator increases with increasing number of applied materials. Therefore, a high thermoelectric performance can be achieved within the large temperature gradient by using a multi-stage system, *i.e.*, segmented and cascaded thermoelectric generators [53]. The Seebeck coefficient of segmented generator Bi_2_Te_3_–PbTe exhibits higher values by about 60%–68% compared to the homogeneous Bi_2_Te_3_ and PbTe materials [54]. According to reference [42], a segmented thermoelectric generator made of Bi_2_Te_2.7_Se_0.3_ and CoSb_3_-based materials is expected to exhibit a thermoelectric performance increased by about 60%, compared to Bi_2_Te_3_- and PbTe-based generators, in a wide temperature range. These improved generators could be used for waste heat recovery. The segmented systems based on SiGe exhibit the improved efficiency, when the hot and cold side temperatures are 1175–1273 K and 573 K, respectively. The segmented generator based on skutterudite alloys has *ZT* ranging to 1.48 at the temperature gradient 300–973 K [55]. The selection of thermoelectric materials strongly affects the generator performance. The materials employed in the fabrication of the segmented thermoelectric generators should be compatible, *i.e.*, similar in terms of thermal conductivity and thermal expansion coefficient, to prevent failure under operating conditions [53,54].

In reference [56], it is proved that the serious disadvantage of alloy based materials is their environmental impact due to the life cycle (e.g., expensive refining processes and useless scrap materials). Processing these materials consumes larger amounts of energy, increasing their cost. Moreover, the recovery and recycling of waste are negligible. Environmentally friendly technologies and materials should eliminate or at least limit the use of heavy metals and reduce the energy consumption of the production processes [41,56].

The stability and toxicity of alloy-based materials is also an issue for many applications. Less toxic inorganic oxides are promising for the thermoelectric energy conversion from waste heat. A number of studies are devoted to this materials, see, e.g., [57,58,59,60,61,62]. Although the thermoelectric performance of oxide-based materials is not as good as tellurides and antimonides alloys, they have better stability and lower negative environmental impact [41]. The oxides with the best thermoelectric properties are Na_x_CoO_2_ and Co_3_Co_4_O_9_, as well as doped CoMnO_3_, doped SrTiO_3_ and doped ZnO [41]. The layered cobaltite based thermoelectric materials may be doped with potassium, bismuth, copper, barium, manganese, strontium, zinc, lanthanum or silver, which influences the figures of merit and thermoelectric efficiency. The value of *ZT* of these materials reaches 0.9 at 1050 K. The strontium titanate based materials exhibit *ZT* up to 0.5 at 1050 K. These oxides also might by doped with different metals or oxides to improve the efficiency. The energy efficiency of mentioned oxide-based materials varies in the range of 5%–15%.

Materials based on of CaMnO_3_ are widely modified by ytrium, lanthanum, cerium, samarium, indium, tin, antimony, lead, and bismuth [57]. The value of *ZT* of CaMnO_3_ and ZnO varies in the range 0.1–0.3 [41]. In reference [58], the In_2_O_3_-based complex oxides and a highly conductive perovskite-type oxide La_1-x_Sr_x_CrO_3_ characterized by *ZT* = 0.45 at 1273 K and *ZT* = 0.14 at 1600 K, respectively, are described. The Ca_2_Co_2_O_5_-type structures exhibit good thermoelectric performance at 700 °C [59]. Other oxides for thermoelectric purposes are LaCoO_3_, LaCuO_4_ and CuAlO_2_ exhibiting the figures of merit (~0.01–0.1) [41].

The big advantage of oxide based materials is their low thermal conductivity (0.1–1.6 W/mK) and the possibility of modification by doping. However, they require sintering at 1000–1300 °C and are thermoelectrically active at high temperatures [41,57]. Thus, oxides may be suitable for high temperature applications.

## 5. Review of Electroactive Polymers for Waste Heat Recovery

Electroactive polymers and polymer composites can supplement the alloy- and oxide-based materials in waste heat recovery from low-grade sources. Polymers have lower negative environmental impact due to their chemical composition, lower manufacturing and processing costs and recycling ability. Polymers are lightweight and may be formed in a variety of shapes, which is important from the application point of view [43,63,64]. Conducting polymers are thermoelectrically active even at an ambient temperature. This is still a relatively new group of materials not fully investigated. Thus, even a small change in their properties may cause a fundamental step forward for their wider application for direct heat recovery and conversion [65].

The applicability of electroactive polymers in thermoelectric devices has been evaluated in a number of studies, see, e.g., references [43,63,66]. The main issue for the practical application of these materials, according to references [43,56,67], is low thermoelectric efficiency.

**Figure 1:**
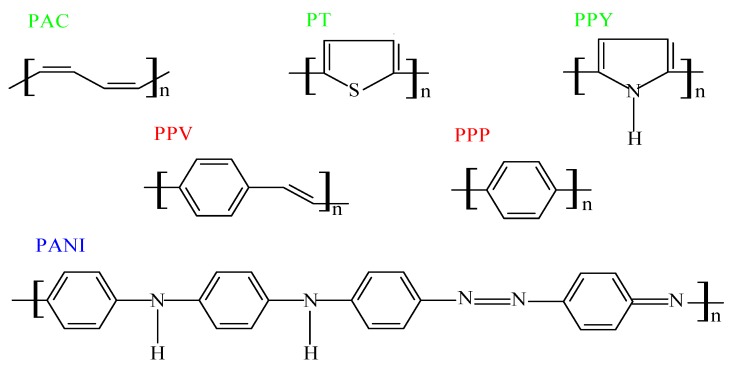
The chemical structures of the chosen conducting polymers.

Currently, approximately 20 structures are known as electroactive polymers [68]. The examples of these materials are polyacetylene (PAC), polypyrrole (PPY), polythiophene (PT), polyaniline (PANI), poly (*p*-phenylene vinylene) (PPV) and poly(*p*-phenylene) (PPP), and their derivatives. The chemical structures of the chosen conducting polymers are presented in Figure 1.

From the electronic point of view, the materials with a larger band gap are characterized by higher Seebeck coefficient. This parameter is significantly bigger for conducting polymers compared to alloys (especially for hydrochloric acid-modified polyaniline) [56]. The charge carriers’ mobility is comparable for these two groups of materials [56,69]. In reference [44], the mobility of the charge carriers *μ* is proposed as one of the criteria of thermoelectric material selection. The thermoelectric effect occurs in materials characterized by *μ* > 0.1 cm^2^/Vs, which is fulfilled for polymers. These electronic parameters influence the macroscopic electric and thermoelectric parameters. The thermoelectric power factor (*α*^2^*σ*) of modified electroactive polymers varies in the range of 10^−8^–10^−3^ W/mK^2^, and the Seebeck coefficient varies from several to hundreds µV/K [63,70,71,72]. The thermoelectric performance of modified polymers is not comparable to the most high-efficient inorganic thermoelectric materials, such as Bi_2_Te_3_. However, the *ZT* values of modified polymers are similar to medium-efficient alloys (e.g., FeSi_2_) [43]. The maximum value of the conductivity of polyacethylene reaches 10^6^ S/cm, which is comparable to metals [68]. For other polymers, lower values of this property are reported [73]. The thermoelectric parameters of electroactive polymers are strongly dependent on the modification methods and the modifier character. Table 3 reports the thermoelectric parameters of the selected electroactive polymers.

The thermoelectric efficiency of all electroactive polymers is similar. The Seebeck coefficient at room temperature ranges from several to several tens of μV/K, and depends not only on the type of polymer and its modification [74,75], but also on the degree of crystallinity and porosity [73]. For example, in the case of PPY, it was found that the values of thermopower for the polymer synthesized by various methods are very similar, but they differ for porous and highly disordered polymers. The Seebeck coefficient depends more strongly on the thermal resistance than on the electrical resistance, and heat is transported by lattice vibrations. Therefore, the thermopower depends more strongly on the crystalline than on the amorphous regions, since the heat flow is limited in a different way than the charge transport [63,73,75].

The results of the investigation presented in reference [76] show that, for polyaniline, polypyrrole and polyacethylene, the highest electrical conductivity is not advantageous for thermoelectric use. Semicrystalline morphology of these compounds makes the charge transport heterogeneous. The electrical conductivity of the crystalline regions can be very high (even metallic), but it is limited by structural barriers in the amorphous areas [75,77]. As mentioned earlier, the Seebeck coefficient is conditioned by different effects; however, the raise in electrical conductivity decreases the value of *α* This dependence is valid for every polymer structure, *i.e.*, oriented films, coatings, and bulks [76]. Therefore, an optimal value of *σ*, balanced with a high value of *α*, should be found. The thermoelectric power factor (*α^2^σ*) combines these parameters. Thermoelectric power factor of iodine-modified polyacetylene and alloy materials is comparable. Other conducting polymers, regardless the modification method, are characterized by the value of (*α^2^σ*) lower by about one order of magnitude [42,63,70,71,72,76,78,79].

According to references [48,75,76,77], polyaniline (PANI) is one of the most suitable electroactive polymers considered for waste heat recovery from low-grade sources, due to the preferred properties and the thermoelectric activity at temperature range of 300–450 K. In comparison to other conductive polymers, this material is inexpensive, its synthesis and processing technologies are simple, and a variety of modification methods is available. Other advantages of polyaniline are the low thermal conductivity (0.2 W/mK), high heat resistance (glass transition temperature above 200 °C, degradation temperature above 300 °C), environmental stability and chemical resistance. Polyaniline can be compounded with materials common in the industry (e.g., metals, glass, plastics, *etc.*) in the form of composites and layer systems [64,67]. The chemical and physical modification of polyaniline may result in the materials with precisely designed properties and characterized by *ZT* value up to 1 × 10^−2^ [42,73,80,81]. It is also the first commercially available conducting polymer. The growing interest in PANI has resulted in a number of scientific studies and products available on the market [48,49,64,81]. In the following part of this section, a review on the methods of improvement of thermoelectric performance of this polymer is presented.

The unmodified polyaniline is an electrical insulator and the thermoelectric phenomenon occurs only in its electroactive derivatives. The common method of increasing the electrical conductivity of polyaniline is the modification by protonic acids. Protonation results in an internal redox reaction and the subsequent change in the electronic structure of the polymer. Transformation from the insulating state to the semi- or even high-conducting salt occurs without a change in the total number of electrons [81]. The electrical conductivity of PANI also strongly depends on the type and amount of a modifier [64,82]. The technology of the synthesis and modification may influence the shape of PANI molecules, its porosity and thus the electrical conductivity or adhesion to the potential substrate [82,83,84]. Appropriately selected acid and the method of its incorporation forces the cylindrical shape of the polyaniline particles, which strengthens the charge transport in the polymer [67,85]. The cylindrical polyaniline was first obtained by modification by *p*-toluenesulfonic acid (TSA) [86,87]. PANI may be protonated by small or large anions of both inorganic and organic acids [82,83,84]. At present, the commonly used inorganic dopants are: hydrochloric acid (HCl), sulfuric acid (H_2_SO_4_) and perchloric acid (HClO_4_). The commonly used organic dopants are benzenesulfonic acid (BSA), *p*-styrene acid (SSA), polyacrylic acid (PAA), camphorsulfonic acid (CSA), dodecylbenzene acid (DBSA) and the others [49,64,67,85]. These modifications can increase the conductivity of the polyaniline even to the metallic values (*σ* ~ 10^2^ S/cm) [85,88,89]. There are many protonation methods available, but the chemical *in situ* method is simple and most popular [49,90]. This method is convenient for obtaining a conducting polyaniline with repeatable molecular weight and thermoelectric properties, and was described in detail in reference [91]. The synthesis method influences the molecular mass, and the larger the molecular mass is, the higher the electrical conductivity, but lower Seebeck coefficients are obtained. The selection of acidic dopant should also compromise between all parameters influencing the value of *ZT*. A number of scientific research works are focused only on improvement of the electrical conductivity of PANI. According to reference [73], the best electrical properties are obtained for polyaniline doped with camphorsulfonic acid while the use of hydrochloric or phosphoric acid results in the material characterized by lower electrical conductivity. According to references [85,88], modification by HCl, HClO_4_, H_2_SO_4_ and H_3_PO_4_ results in conductivity values of 200–300 S/cm at a room temperature. However, as it was mentioned earlier, modification should be focused on all parameters influencing the thermoelectric efficiency. In reference [49], it was pointed out that the *ZT* value for PANI-HCl reaches 2.67 × 10^−4^ at 423 K. According to reference [89], with the use of *p*-toluenesulfonic acid, a material characterized by the value of *ZT* ≈ 10^−5^ may be obtained. The use of acetic acid results in polyaniline characterized by the value of *ZT* ≈ 10^−8^ (at 300 K). The polyaniline obtained by doping with a boric acid exhibits the electrical conductivity of 10^−4^ S/cm and thermoelectric power factor (*α^2^σ*) 0.64 μW/mK^2^. However, this material does not exhibit the cylindrical structure [92]. Protonation of polyaniline by phosphoric acid results in the material characterized by the value of thermopower equal to *α* = 7.6 μV/K [93]. By the protonation by hydrochloric acid, polyaniline characterized by *α* = 177 μV/K and *ZT* = 6.63 × 10^−3^ (at a room temperature) may be obtained [88].

In order to further improve the thermoelectric efficiency of polyaniline protonated by acids, conductive additives may be employed. A number of studies [43,64,65,68,94,95,96,97] suggest that the presence of inorganic powder filler may increase the electrical conductivity of polyaniline to the values comparable to alloy-based materials. Powder fillers should not negatively influence the lightweight, flexibility and processabitlty of polyaniline [98]. The fillers that may be applied are noble metals (e.g., gold, platinum, palladium and silver), base metals (copper, nickel, cobalt, iron, zinc, bismuth, manganese, *etc.*) either semimetals (e.g., selenium and aluminum) and their oxides or alloys, as the non-metallic modifiers sulfuric and carbon fillers (e.g., nanotubes, graphene, carbon black and graphite) can be applied [99]. A number of studies show the possibility of polyaniline modification by different thermoelectrically active materials, e.g., bismuth telluride [67], bismuth [48], and scutterudite [96].

The composite of bismuth telluride and polyaniline protonated by hydrochloric acid (PANI-HCl/Bi_2_Te_3_) exhibits the value of Seebeck coefficient close to Bi_2_Te_3_, but the electrical conductivity is almost equal to the unfilled PANI-HCl. Furthermore, as a synergistic effect of Seebeck coefficient and electrical conductivity, the power factor (*α^2^σ*) of the composite is lower than of both pristine components Bi_2_Te_3_ and PANI-HCl [67]. Polyaniline, as a minor component, can be added to Bi_0.5_Sb_1.5_Te_3_ in an amount of 1–10 vol %. The thermoelectric performance of such a composite will depend on the host (inorganic) material [48]. In turn, the presence of large quantities of skutterudite microfibers does not significantly change the Seebeck coefficient of the polymer matrix [96]. The composite of polyaniline and bismuth nitrate (PANI/Bi(NO_3_)_3_) shows the structure of nanorods and a charge transport mechanism consisting of metallic conductivity and tunneling. The incorporation of 10 wt % of molybdenum oxide (MoO_3_) positively affects the charge transport in the polyaniline and increases the value of the electrical conductivity up to 10^−3^ S/cm [100].

Particles of conductive additives, such as Bi_2_Te_3_, Bi_0.5_Sb_1.5_Te_3_, Bi(NO_3_)_3_, are often micron sized. Thus, they do not affect the quantum effects. In addition to large particle size, such complex materials are difficult to produce [48]. For this reason, the polyaniline is also being modified by smaller and/or less complex conducting additives [64,99]. A fine filler interacts strongly with the polymer. The PANI composites with nanofillers exhibit higher electrical conductivity compared to microfillers. Nanoparticles incorporated properly into the polymer matrix will improve thermoelectric performance as well as a mechanical and thermal resistance. The composites of polyaniline and nano-metals can combine the advantages of these two groups of components [98]. Metallic nanoparticles incorporated into polyaniline build a conducting network in the matrix. This network positively influences the thermoelectric, thermal and electric properties on the atomic (quantum) scale. In such a composite, polyaniline acts as a mechanical and electrical binder for the metal [48]. The application of nanoparticles allows for reducing the filler load in comparison to micron sized additives [48,101,102,103].

Hostler *et al.* [48] determined the dependence between the thermoelectric properties and the filler amount for the composite of polyaniline protonated by camphorsulfonic acid and nanobismuth (PANI-CSA/Bi). Increasing the nanofiller load (up to 10 vol %) positively affects the electric conductivity of the composite. In the presence of Bi, the arrangement of the polyaniline particles increases, which additionally improves charge transport. Further increase of Bi load negatively influences the electrical conductivity, which may be related to the scattering effect of the nanoparticles. The composite with the content of 50 vol % this metal is an electrical insulator, thus the Seebeck effect does not occur. The thermal conductivity of the composite with Bi content of 5–10 vol % rapidly drops. From the macroscopic point of view, this value should raise with increasing bismuth ratio, since it is significantly bigger for the pristine metal than the unfilled PANI-CSA. However, it is not known whether the properties for the pristine bulk and nanoparticles of bismuth are equal. Additionally, the polymer-metal interphase significantly affects the thermal conductivity and may reduce its value by the dissipation effect. The Seebeck coefficient of the composites varies slightly for low bismuth content (up to 10 vol %.), but, regardless of the filler load, the value of *α* is closer to the value of the unfilled polymer than the pristine metal. *ZT* values of PANI-CSA/Bi composite present a similar trend, *i.e.*, slight increase at low Bi content, mainly due to increased electrical conductivity. At higher Bi load, the value of *ZT* decreases. The *ZT* values of the composites are closer to the unfilled PANI-CSA than for Bi, and equal to *ca*. 1 × 10^−3^ [48]. It is also possible to apply carbons (*i.e.*, graphite, carbon black, carbon nanotubes, *etc.*) as fillers for the polyaniline. The composites characterized by electrical conductivity up to 10 S/cm can be obtained, by incorporation of 10–50 wt % of graphite [104]. In the composites with high (over 90%) graphite content, polymer improves the charge transport between the clusters of the carbon host. Such a material exhibits superior conductivity in comparison to the pristine components [105]. However, it is very difficult to process.

According to references [98,106,107], only the use of acid doped conducting polyaniline results in delocalization or the percolation path in the composite with powder fillers. The composite of non-protonated polyaniline with conducting additive is an electrical insulator for a wide range of the filler load. The charge is concentrated in the filler particles surrounded by the insulating polymer matrix.

As mentioned earlier, the thermoelectric properties of polyaniline based materials are strongly dependent not only on the type and amount of the modifier, but also on the manufacturing and processing methods. Thus, the synthesis and processing technologies should be also considered as modification methods. The variety of possibilities to obtain shapes (thin layers, fibers, bulk products, *etc.*) gives the opportunity of polymer spreading on different waste heat-dissipating surfaces (flat, curved and finned surfaces, *etc.*). According to reference [43], the thin film of PANI has the *ZT* *ca.* 10^−2^ and may be obtained by the spin-coating method. Thin films made from modified polyaniline exhibit values of *ZT* of 2–3 orders of magnitude higher than the bulk products with the same composition. The stretched structure positively affects the values of *ZT*, whereas a higher anisotropy and expanded chains conformation increases the electrical conductivity [107]. The multilayer system will additionally improve the electronic properties and thermoelectric performance of PANI-based materials. Polyaniline based coatings may be manufactured using different methods, *i.e.*, spin-coating, electrochemical deposition, dip-coating, drop-coating, thermal evaporation, the Langmuir–Blodgett technique and self-assembly [43,66,108,109,110]. Depending on the selected method the products will differ in structure, chemical resistance or mechanical and electrical properties. Polyaniline fibers with different diameter and length may be prepared e.g., from the solution or by electrospining method [111,112]. The fibers made from polyaniline (or polyaniline blends) may be further used as a raw material for mats and textiles [111].

However, according to reference [49] and the authors’ own experiences [107], the polyaniline bulk products also have a great potential for thermoelectric heat recovery. The three-dimensional products may be formed using classical processing methods (*i.e.*, press molding, extrusion and injection). According to references [113,114], these processing technologies may negatively influence the electrical conductivity of polyaniline. This problem may be eliminated by the blend technology and compounding polyaniline with classical plastics [115,116].

In summary, polyaniline based materials should be considered as a future supplement for inorganic materials for waste heat recovery from low-grade sources, where the alloy- and oxide-based materials are not fully active. However, the thermoelectric performance of these materials should be improved and a number of available modification methods provides such possibilities. Not only the chemical modification (via protonation acids and conducting additives), but also the physical techniques (via processing) are available. It is possible to employ polyaniline based materials as the homogenous material or a compound of a composite. Combining of polyaniline into segmented and multilayered systems is an idea for future works.

## 6. Conclusions

A review on thermoelectric materials for direct waste heat recovery gives the following conclusions:

- Industrial and distributed waste heat sources can be divided, by temperature range, into three groups: high-grade, medium-grade and low-grade. The heat source temperature range is the main criterion when selecting the appropriate recovery technology.
- Present waste heat recovery technologies, such as ORCs, Kalina systems and Stirling engines, are not suitable for direct waste heat recovery from solid surfaces of installations and machines, and are mechanically complicated.
- A comparison between thermoelectric materials and currently used waste heat recovery technologies highlights advantages of the former (lack of moving parts, working fluids, *etc.*). Thermoelectric materials may be employed for direct waste heat recovery. The heat source temperature range is an important parameter when selecting the thermoelectric material.
- Alloy- and oxide-based materials are suitable for thermoelectric waste heat recovery from medium- and high-grade sources.
- Tellurium-, antimony- and germanium-based alloys achieve the largest thermoelectric performance.
- The thermoelectric efficiencies and temperature ranges of alloy-based materials can be improved by compounding into segmented generators.
- Oxide-based materials are less efficient than alloys, are more toxic, and are worse for the environment. However, they are more stable.
- Oxide-based materials with the highest thermoelectric performance are Na_x_CoO_2_, Co_3_Co_4_O_9_, doped CoMnO_3_, doped SrTiO_3_ and doped ZnO.
- Electroactive polymers show potential for direct waste heat recovery from low-grade sources, and polyaniline based materials are the most promising due to their good chemical and thermal stability and low manufacturing costs.
- The thermoelectric efficiency of polyaniline is poor compared to inorganic materials. However, there are a number of chemical and physical modification methods available for improving its properties.
- Polyaniline exhibits thermoelectric performance in the low temperature range, where inorganic materials are not fully active.
- Polyaniline based materials may be formed in a variety of shapes and combined with other materials. Such material properties are particularly advantageous direct waste heat recovery from solid surfaces, since the material can be spread on surfaces of different geometries, e.g., flat, curved.

In summary, thermoelectrics are promising materials for direct waste heat recovery. The working temperatures of all three types of materials discussed overlap with the temperature ranges of waste heat sources of various grades. Research on thermoelectric waste heat recovery should focus on electroactive polymers dedicated to low-grade sources. Polyaniline based materials for waste heat recovery should be fine tuned by raising their thermoelectric performance and combining them with other materials into multilayer or segmented systems.

## Figures and Tables

**Table 1:** The temperature ranges of the selected waste heat sources [3,5].

Industrial/Distributed Energy Conversion Process	Heat Carrier	Temperature Range (°C)
Power plant	Exhaust gases	250–1200
	Cooling mediums	40–150
	Solid and liquid waste	40–200
	Waste steam	150–300
	Hot elements	40–400
Chemical plant	Process gases	100–600
	Liquids	40–200
	Hot elements	40–300
Food processing plant	Liquids	40–100
	Cooling air	50–100
	Hot elements	40–400
Steel-mill	Exhaust gases	250–1200
	Process gases	300–1400
	Cooling mediums	40–150
	Solid and liquid waste	40–200
	Hot elements	40–700
Road and rail transport	Exhaust gases	500–1100
	Coolants	40–100
	Hot elements	40–500
Housing and industrial building	Flue gases	150–300
	Liquids	40–90

**Table 2:** The thermoelectric parameters of selected semiconductors and metals [50,51].

Material	*σ* (1/Ω∙cm)	*α* (µV/K)	*α*^2^*σ* (W/mK^2^)	*k* (W/mK)	*Z* (K^−1^)
Bi_2_Te_3_	1000.0	200.00	4.0 × 10^−3^	1.60	3.0 × 10^−3^
PbTe	450.0	20.00	2.6 × 10^−3^	2.00	1.2 × 10^−3^
SiGe *p*-type	758.0	144.00	1.6 × 10^−3^	4.80	3.3 × 10^−4^
SiGe *n*-type	990.0	−136.00	1.8 × 10^−3^	4.45	4.1 × 10^−4^
Cu	580,000.0	1.83	1.9 × 10^−4^	398.0	4.8 × 10^−7^
Ni	138,889.0	−19.50	5.3 × 10^−3^	90.50	5.9 × 10^−5^
Ti	23,810.0	9.10	2.0 × 10^−4^	21.90	9.1 × 10^−6^

*α* (V/K) is the Seebeck coefficient; *σ* (1/Ω∙cm) is the electrical conductivity; *α^2^σ* (W/mK^2^) is the thermoelectric power factor; *k* (W/m∙K) is the thermal conductivity; *Z* (K^−1^) is the thermoelectric figure of merit.

**Table 3:** The thermoelectric parameters of the selected electroactive polymers.

Polymer	Modifier	*σ* ((Ω∙cm)^−1^)	*α* (µV/K)	*α^2^σ* (W/mK^2^)
Polyacethylene (PAC)	–	6405	20.6	2.7 × 10^−4^
	I *	60,000	15.0	1.3 × 10^−3^
Polyaniline (PANI)	–	18	3.0	1.6 × 10^−8^
	CSA **	200	10.0	2.0 × 10^−6^
Polypyrrole (PPY)	–	26	5.0	6.5 × 10^−8^
	PANI	15	7.0	7.4 × 10^−8^

* I—iodine; ** CSA—camphorsulfonic acid.
